# Cognitive Style and Frame Susceptibility in Decision-Making

**DOI:** 10.3389/fpsyg.2018.01461

**Published:** 2018-08-10

**Authors:** David R. Mandel, Irina V. Kapler

**Affiliations:** ^1^Intelligence Group, Intelligence, Influence and Collaboration Section, Defence Research and Development Canada, Toronto, ON, Canada; ^2^Department of Psychology, York University, Toronto, ON, Canada

**Keywords:** framing effect, risky choice, Asian disease problem, cognitive style, individual differences

## Abstract

The susceptibility of decision-makers’ choices to variations in option framing has been attributed to individual differences in cognitive style. According to this view, individuals who are prone to a more deliberate, or less intuitive, thinking style are less susceptible to framing manipulations. Research findings on the topic, however, have tended to yield small effects, with several studies also being limited in inferential value by methodological drawbacks. We report two experiments that examined the value of several cognitive-style variables, including measures of cognitive reflection, subjective numeracy, actively open-minded thinking, need for cognition, and hemispheric dominance, in predicting participants’ frame-consistent choices. Our experiments used an isomorph of the Asian Disease Problem and we manipulated frames between participants. We controlled for participants’ sex and age, and we manipulated the order in which choice options were presented to participants. In Experiment 1 (*N* = 190) using an undergraduate sample and in Experiment 2 (*N* = 316) using a sample of Amazon Mechanical Turk workers, we found no significant effect of any of the cognitive-style measures taken on predicting frame-consistent choice, regardless of whether we analyzed participants’ binary choices or their choices weighted by the extent to which participants preferred their chosen option over the non-chosen option. The sole factor that significantly predicted frame-consistent choice was framing: in both experiments, participants were more likely to make frame-consistent choices when the frame was positive than when it was negative, consistent with the tendency toward risk aversion in the task. The present findings do not support the view that individual differences in people’s susceptibility to framing manipulations can be substantially accounted for by individual differences in cognitive style.

## Introduction

Literature on risky choice shows that, in general, people are susceptible to a wide range of framing effects. Such effects signal incoherence in decision-making because they ostensibly violate the description invariance principle, which states that mere re-descriptions of events that do not alter their extension should, likewise, not alter people’s choices. The description invariance principle is one of the least controversial coherence principles undergirding rational choice theory, and thus violations of it are regarded as prima facie evidence of irrationality in human decision-making (Arrow, 1982; Tversky and Kahneman, 1986).

The most frequently studied type of framing effect involves re-describing the possible outcomes of two alternative options in terms that are meant to either emphasize gain (positivity) or loss (negativity). Tversky and Kahneman’s (1981) Asian Disease Problem (ADP) provides a seminal demonstration of the manipulation. All participants first read the following:

Imagine that the United States is preparing for the outbreak of an unusual Asian disease, which is expected to kill 600 people. Two alternative programs to combat the disease have been proposed. Assume that the exact scientific estimates of the consequences of the programs are as follows:

Participants in the positive-framing condition chose between the following options:

If Program A is adopted, 200 people will be saved.

If Program B is adopted, there is 1/3 probability that 600 people will be saved, and 2/3 probability that no people will be saved.

Participants in the negative-framing condition instead chose between these options:

If Program C is adopted, 400 people will die.

If Program D is adopted, there is 1/3 probability that nobody will die, and 2/3 probability that 600 people will die.

Seventy-two percent chose the certain option (A) under positive framing, whereas 78% chose the uncertain option (D) under negative framing.^1^

The framing effect demonstrated using the ADP and problem isomorphs—namely, the tendency to make risk-averse choices given positive frames (option-A choices) and risk-seeking choices given negative frames (option-D choices)—is highly replicable and tends to yield small to moderate effect sizes (for meta-analyses, see Kühberger, 1998; Piñon and Gambara, 2005). Although much of the literature on framing effects and the ADP, in particular, has focused on nomothetic patterns of choice, it is evident that susceptibility to frame-consistent choices exhibits individual differences, which theoretical accounts of framing tend to neglect. For instance, individual differences in the linguistic interpretation of numerical quantifiers in the two options of the ADP influence the proportion of samples showing the standard framing effect. Mandel (2014, Experiment 3) found that the standard framing effect was observed among participants who interpreted the numeric quantifier in the certain option as a lower bound (i.e., meaning *at least*), but it was not observed among participants who interpreted the same quantifiers as meaning an exact value (i.e., either *exactly* 200 will be saved in the positive frame or *exactly* 400 will die in the negative frame). A minority of participants who interpreted the same quantifiers as representing an upper bound (i.e., *at most*…) actually showed a reversed framing effect.

By far, however, most attention to individual differences in susceptibility to framing on choice has focused on variations in people’s cognitive style. The interest, in part, would seem to stem from a more recent view of such effects as relying on fast and intuitive “System 1” reasoning processes rather than slower, analytic “System 2” reasoning processes (Stanovich and West, 2000; De Martino et al., 2006; Evans, 2008, 2010; Kahneman, 2011). Consistent with this view, some studies find that requiring people to thoughtfully consider problem options (i.e., to shift from System 1 reasoning to the more deliberate and effortful, System 2 reasoning) attenuates framing effects. For example, Takemura (1994) asked participants to either write an open-ended justification for their choices in the ADP or simply choose between the two programs. In the high-elaboration condition, the framing effect was eliminated. Likewise, Almashat et al. (2008) found that deeper deliberation in medical decision-making, achieved by asking participants to list advantages and disadvantages of each treatment option prior to making a choice, reduced framing effects (see also Miller and Fagley, 1991; Sieck and Yates, 1997).

If situational manipulations that affect the degree of deliberateness in decision-making can moderate framing effects, then perhaps individual differences in cognitive-style measures that track deliberateness in thinking might also predict susceptibility to frame-consistent patterns of choice. One such hypothesis is that need for cognition (NFC) moderates framing effects. NFC measures the extent to which individuals enjoy engaging in effortful thinking (Cacioppo et al., 1984). Several studies have explored the relationship between NFC and framing effects. For example, Smith and Levin (1996) found that participants high in NFC showed no framing effect on multiple decision tasks, whereas participants low in NFC showed framing effects (see also Carnevale et al., 2011). However, Simon et al. (2004) in a between-subjects design found that being high in NFC alone was insufficient to eliminate framing effects. Only participants who were high in NFC and high in self-rated math or engaged in deep thinking showed reduced susceptibility to framing effects (also see Covey, 2014). LeBoeuf and Shafir (2003) asked participants to answer a number of framing problems as well as provide justifications for their responses. Although NFC did not moderate the framing effect when participants were exposed to only one frame in a between-subjects design, participants high in NFC were more likely than low-NFC participants to make consistent choices across frames when presented with both frames in a within-subjects design. LeBoeuf and Shafir (2003) posited that NFC increased consistency detection across frames but did not diminish the framing effect when it was impossible for participants to verify their consistency in choice. However, Levin et al. (2002) did not find a relation between NFC and framing even when utilizing within-subjects designs (participants answered both frames options separated by 1 week in time). Likewise, Peters and Levin (2008) and Mandel (2014, Experiment 1) did not find evidence that NFC moderated the framing effect.

A related cognitive-style measure that has been explored as a possible moderator of the framing effect is actively open-minded thinking (AOT). AOT involves a willingness to evaluate evidence that goes against one’s beliefs, and openness to considering alternative perspectives (Baron, 1985, 1993; Haran et al., 2013; Baron et al., 2015). AOT and NFC are positively correlated (West et al., 2008, 2012; Haran et al., 2013). AOT has been positively associated with accuracy in forecasting (Tetlock, 2005; Mellers et al., 2015) and other probabilistic judgment tasks (Haran et al., 2013). Actively open-minded thinkers are also less prone to biases, including myside bias (Baron, 2008; West et al., 2008), belief bias, framing, and base-rate neglect (West et al., 2008; Toplak et al., 2011, 2017).

A third cognitive-style measure that might be expected to index the degree of System 2 reasoning is the Cognitive Reflection Test (CRT; Frederick, 2005), which measures individuals’ abilities to suppress incorrect intuitive answers and answer correctly. CRT performance is positively related to AOT (e.g., Toplak et al., 2014; Baron et al., 2015) and to performance on risky choice tasks (Cokely and Kelley, 2009). CRT is also negatively related to a wide range of cognitive biases in judgment and decision-making (e.g., Toplak and Stanovich, 2002; Campitelli and Labollita, 2010; Toplak et al., 2011; Baldi et al., 2013). The evidence regarding the relation between framing susceptibility and CRT is mixed, however, with some literature reporting positive relations (Oechssler et al., 2009; Noori, 2016) and other literature reporting no relation (Toplak et al., 2014; Aczel et al., 2015). There is also disagreement about what CRT measures. Sinayev and Peters (2015) posit that CRT mainly taps numeracy skill, whereas Pennycook and Ross (2016) examined evidence showing that CRT predicted a wide range of variables not attributable to numeracy. Szaszi et al. (2017) concluded that both numeracy and cognitive reflection (indicative of System 2 reasoning) account for CRT performance. However, item analysis of the CRT showed that only one of the three items (the bat and ball problem) had faster response time when the answer was the intuitive incorrect response than when it was the correct response (Stupple et al., 2017). Moreover, even in that case, the effect was small. Such findings raise doubt about the extent to which performance on the measure captures “counter-deliberate” cognitive miserliness.

Lipkus and Peters (2009) posited that numeracy has a number of functions, such as facilitating assessment of likelihood and value, improving interpretation and acceptance of numerical data, encouraging information seeking and greater depth of processing. Yet numeracy shows a mixed pattern of evidence in studies on framing. In Peters et al. (2006, Study 1), less numerate participants showed larger framing effects when asked to rate the quality of students’ work that was presented either as percent correct (74%) or percent incorrect (26%) on an exam (see also Garcia-Retamero and Galesic, 2010; Peters, 2012). Likewise, Peters and Levin (2008) found that less numerate participants showed larger framing effects on choice than more numerate participants. However, Peters et al. (2011) found that numeracy did not moderate framing effects on a task that assessed medication risk. Whereas numeracy is often *objectively* measured in terms of performance skill (e.g., Lipkus et al., 2001; Weller et al., 2013), it can also be measured using a Subjective Numeracy Scale (SNS), which taps individuals’ preferences for processing numbers and graphical information over words (Fagerlin et al., 2007). Individuals lower in subjective numeracy had more negative emotional reactions to numbers and were less motivated and/or confident in numeric tasks (Peters and Bjalkebring, 2015). Compared with objective numeracy scales that require participants to complete calculations, SNS has several advantages (Fagerlin et al., 2007; Kee and Liang, 2015): it takes less time to complete, participants find it more enjoyable, less stressful and less frustrating, and there is direct (Zikmund-Fisher et al., 2007) and indirect (Weller et al., 2013) evidence that SNS is a good approximation of objective numeracy. For instance, moderate correlations (in the 0.4–0.7 range) between objective and subjective numeracy scales also have been reported (Fagerlin et al., 2007; Peters and Bjalkebring, 2015; Gamliel et al., 2016). Moreover, unlike objective numeracy, SNS cannot be exploited by use of a calculator, an issue of concern in online studies such as those we report in this article.

### The Present Research

The present research builds on prior work examining how cognitive-style measures relate to susceptibility to framing. Although several studies have examined this issue, there has been little attempt to jointly examine cognitive-style measures as predictors of frame susceptibility. Multi-measure analyses are critical, however, because measures such as NFC, AOT, CRT, and SNS share considerable variance.^2^ Several studies also have binned participants into high versus low categories based on median splits. This is usually a poor statistical method of analysis because it adds error, reducing statistical power and increasing the likelihood of Type II errors in many cases (Humphreys and Fleishman, 1974; Cohen, 1983). Moreover, in other cases, dichotomizing continuous variables can lead to spurious statistical significance or Type 1 errors (Maxwell and Delaney, 1993). As well, most studies have taken a rather coarse moderator approach to analysis in which the cognitive-style measure, partitioned into high versus low scores, is crossed with a framing manipulation. Evidence of moderation takes the form of showing that the framing effect is smaller in one group than the other. This method is theoretically imprecise because significant effects of framing do not necessarily conform to theoretical expectation. Consider a hypothetical case of an ADP experiment using a large sample in which 90% of participants in the positive-frame condition choose option A, whereas “only” 75% choose option A in the negative-frame condition. The framing effect may be significant, yet most participants who encountered the negative frame would not have responded as predicted by prospect theory (Kahneman and Tversky, 1979) or other theories of framing, such as the explicated valence account (Tombu and Mandel, 2015; also Wallin et al., 2016) or the fuzzy trace account (Reyna and Brainerd, 1991), all of which make the same predictions for the standard ADP (but which diverge in prediction as well as explanation under other task conditions).

Therefore, in the present research, we examine frame susceptibility in a theoretically precise manner. Participants are said to be frame susceptible or to have made frame-consistent choices if and only if they choose the certain option given a positive frame or else they choose the uncertain option given a negative frame. This operationalization of frame-consistent choice thus requires the demonstration of what has been referred to in previous literature as bidirectional framing effects (Wang, 1996) or meeting the reference distribution criterion (Mandel, 2001). In two experiments, we test various predictive models of frame susceptibility. To the extent that these predictors significantly predict frame susceptibility, it would therefore provide more compelling evidence of moderation of framing effects than we have seen in previous studies. In addition to examining NFC, AOT, CRT, and SNS, we include participants’ sex and age in our analysis. This is important because sex has been shown to moderate framing effects (with females showing stronger framing effects; see Piñon and Gambara, 2005 for a meta-analysis) and there is some evidence for sex differences on measures such as CRT (Frederick, 2005), numeracy (objective: Peters et al., 2011; subjective: Peters and Bjalkebring, 2015) and AOT (Toplak et al., 2017) measures, with males scoring higher than females. In terms of age, there appears to be age-related stability in susceptibility to framing (Mayhorn et al., 2002; Rönnlund et al., 2005; Strough et al., 2011). More generally, description-based (as opposed to experience-based) tasks like the ADP tend to show negligible age effects (Mata et al., 2011). Nevertheless, we statistically control for age in the predictive models tested in the present research as a precautionary measure.

Furthermore, we took methodological precautions that have been overlooked in most previous framing research. We manipulated option order as recommended by Fagley and Miller (1997). In most studies, the certain option is presented first, yet Bar-Hillel et al. (2014) have shown a “reachability bias” in response-option selection favoring the option presented first. Kühberger and Gradl (2013) found that although option order had no effect on choice in the positive-frame condition, a greater proportion of participants in the negative-frame condition chose the uncertain option when it was presented after the certain option than when it was presented initially, contrary to the reachability bias. However, using a substantially larger sample, Schwitzgebel and Cushman (2015) found evidence of an order effect in the ADP consistent with reachability bias. In the negative frame, a significantly greater proportion of participants chose the uncertain option when it was presented first. Likewise, in the positive frame a significantly greater proportion chose the certain option when it was presented first. Thus, the standard presentation (certain option first) might overestimate frame susceptibility in the positive-frame condition and underestimate it in the negative-frame condition.

As noted earlier, Mandel (2014) found that the modal interpretation of numerical quantifiers in the certain option was lower-bounded (i.e., “at least *n* will be saved/will die”). On the basis of those and other findings (e.g., Halberg and Teigen, 2009; Teigen and Nikolaisen, 2009), Mandel (2014) recommended that researchers using the ADP make explicit that the numerical quantifiers are intended to be treated as exact values, in order to increase the likelihood that the assumption of extensional equivalence between reframed options is valid. Accordingly, the present research used an isomorph of the ADP, which stated that the value in the certain option presented was *exactly* that value. Finally, following earlier studies (e.g., Mandel, 2001; Tombu and Mandel, 2015), we examine the effects of our predictors on participants’ binary choices as well as on a bi-directional strength of preference measure that weights the chosen option by the degree to which that option is judged preferable to its alternative.

## Experiment 1

### Materials and Methods

Prior to the initiation of this research, it was reviewed and approved by the York University’s Ethics Review Board and deemed to be in conformance with the standards of the Canadian Tri-Council Research Ethics guidelines. Electronic written informed consent was obtained from all participants, who were also debriefed following the experiment.

We recruited 201 undergraduate students enrolled in a first-year psychology course at York University. Students were awarded course credit for participation. Mean age of the sample was 21.7 years (*SD* = 4.2), and 74.2% were female. Eleven participants were removed from the analysis because the integrity of the collected data was low. Specifically, participants were removed due to (a) unreasonable time for completion (over 24 h, or under 4 min), (b) below 50% self-reported English proficiency (current range 55–100, *M* = 92.8, *SD* = 10.7) or (c) unwarranted age for a university sample (e.g., one participant reported being 10 years old). Data from the remaining 190 participants were analyzed.

Experiment 1, which took 15 min of average to complete (*SD* = 12.50), was conducted online using the Qualtrics survey software system. First, participants completed electronic informed consent, where, after reading study details, they had the choice of proceeding with the study or quitting. By proceeding they gave their consent for participation. Participants then were asked to fill out a short demographic questionnaire that asked about age, sex, native language, and self-reported English proficiency.

Next, participants were randomly assigned to one of the four experimental conditions in a 2 (Frame: positive, negative) × 2 (Order: certain-option first, uncertain-option first) between-subjects design. They were then presented with a modified financial version of the ADP taken from Tombu and Mandel (2015). Unlike the original ADP, the numeric quantifiers in the two options were qualified with the term *exactly* to increase the likelihood that the two frames would be represented by participants as extensionally equivalent (Mandel, 2014). As well, to reinforce understanding that the numeric quantifiers referred to exact amounts, they were dually described in terms of number and a fraction of the total amount in question. Specifically, in the positive-frame condition, participants were presented with the following description:

Imagine that a financial investment of yours worth $600 has gone sour. If you do nothing you will lose all of it for sure. However, you have two options that are not as bad:

If you choose option A, you will *keep* exactly one-third ($200) of your investment for sure.

If you choose option B, you have exactly a one-third chance of *keeping everything* ($600) and a two-thirds chance of *keeping nothing* ($0).

In the negative-frame condition, the options were alternatively described as follows:

If you choose option A, you will *lose* exactly two-thirds ($400) of your investment for sure.

If you choose option B, you have exactly a one-third chance of *losing nothing* ($0) and a two-thirds chance of *losing everything* ($600).

As in the ADP, the first task was to choose one of the two options. Subsequently, participants were asked how much they preferred their chosen option over the other option on a scale from 1 (no preference) to 7 (strong preference). The choice and preference measures were recoded as follows for the purpose of data analysis. Choices were coded as frame-consistent if and only if they were (a) certain-option choices made in the positive-frame condition or (b) uncertain-option choices made in the negative-frame condition. Next, frame-consistent choices were dummy coded 1, whereas frame-inconsistent choices were dummy coded -1, and these values were multiplied by the strength of preference scores to provide a preference-weighted measure of frame consistency.

After the decision-making task, participants completed the four cognitive-style measures described earlier in the following order: CRT (Frederick, 2005); SNS (Fagerlin et al., 2007); AOT (Baron, 1993; Haran et al., 2013); and NFC (Cacioppo et al., 1984). Using the 3-item version of the CRT, participants were presented with multiple-choice response options. For instance, one problem is, “A bat and a ball cost $1.10 in total. The bat costs $1 more than the ball. How much does the ball cost?” In earlier studies, participants tend to choose the intuitive answer of 10 cents, even though the correct response is 5 cents. CRT scores were obtained by summing the number of correct responses to the three questions, *M*_sum_ = 0.69, *SD* = 1.0, and Cronbach’s alpha = 0.71. Participants completed the 8-item SNS (Fagerlin et al., 2007), responding on a 6-point scale (1 = Not at all good, 6 = Extremely good). Examples include, “How often do you find numerical information to be useful?” and “How good are you at figuring out how much a shirt will cost if it is 25% off?” SNS scores were obtained by averaging the responses of the eight items, *M* = 3.98, *SD* = 0.94, and Cronbach’s alpha = 0.79. AOT was measured using the 7-items used in Haran et al. (2013) and using a 7-point scale (1 = completely disagree, 4 = neutral, and 7 = completely agree). Examples of AOT items include: “People should revise their beliefs in response to new information or evidence” and “Intuition is the best guide in making decisions” (reversed scored). AOT scores were obtained by averaging responses to the seven items, *M* = 4.88, *SD* = 0.80, and Cronbach’s alpha = 0.67. Finally, we used the 18-item version of the NFC scale (Cacioppo et al., 1984). Participants responded to the item statements on 9-point scales (4 = very strongly agree, 0 = neutral, -4 = very strongly disagree). Examples include, “I find satisfaction in deliberating hard and for long hours” or “It’s enough for me that something gets the job done; I don’t care how or why it works” (reverse coded). NFC scores were obtained by averaging the responses of the 18-items, *M* = 5.58, *SD* = 0.94, and Cronbach’s alpha = 0.85.

In general, then, scale reliabilities were above 0.7 (i.e., a conventional cutoff) with the exception of AOT, which was close and not particularly unusual. For instance, Baron et al. (2015) reported a scale reliability of 0.67 for AOT. Haran et al. (2013) did not report scale reliabilities for AOT but Uriel Haran shared the raw data from Experiments 1–3 with us and we verified that the scale reliabilities were 0.70, 0.76, and 0.75 for Experiments 1–3 in Haran et al. (2013), respectively.

We also tested whether any of the cognitive-style measures may have significantly differed across the two framing conditions. Although the differences were non-significant for SNS, AOT, and NFC, CRT scores were significantly higher in the positive-frame condition (*M* = 0.88, *SD* = 1.10) than in the negative-frame condition (*M* = 0.49, *SD* = 0.86), *t*(188) = 2.71, *p* = 0.007.

### Results and Discussion

Out of 190 participants, 118 (62%) made frame-consistent choices (see **Supplementary Data Sheet S1** for data from Experiments 1 and 2). This proportion significantly exceeds chance, as the binomial probability of finding 118 or more frame-consistent choices is 5.21 × 10^-4^ (in more conventional terms, *p* < 0.001). Recall that frame-consistent choices were coded as 1 and frame-inconsistent choices were coded as -1. The expected value based on chance selection is 0, and the observed mean value is 0.19 (*SD* = 0.98). By Cohen’s (1992) criteria, this corresponds to a small effect size, *d* = 0.20.

It is of theoretical interest to compare the difference between this effect size estimate and one obtained from a traditional between groups test of the framing effect. Given that the former is more conservative (i.e., it requires choosing the certain option in the positive-frame condition or the uncertain option in the negative-frame condition), we expect that the effect size of the traditional effect will be larger. Indeed, this is the case. Coding selections of the certain and uncertain options as 1 and -1, respectively, reveals a mean value of 0.02 (*SD* = 1.01) in the negative-frame condition and a mean value of 0.49 (*SD* = 0.88) in the positive-frame condition, *t*(188) = 3.43, *d* = 0.50, *p* = 0.001. Thus, by applying the stricter (theory-constrained) criterion, the effect size is more than halved.

Recall that Schwitzgebel and Cushman (2015) found that, in line with the reachability bias, frame-consistent choice was more probable when the frame-consistent option was presented first. To test for this effect, we computed a binary measure of whether the frame-consistent choice was presented first or second. The proportion of frame-consistent choices when the frame-consistent option was presented first (0.67) was not significantly greater than the proportion of such choices when the frame-consistent option was presented second (0.58), *p* = 0.23 by Fisher’s two-sided exact test. Furthermore, Goodman and Kruskal’s tau, which measures the fraction of variability in the categorical variable *y* (frame-consistent choice) that can be explained by the categorical variable *x* (whether the frame-consistent option was presented first), was miniscule, τ = 0.008. Therefore, we did not replicate the aforementioned finding by Schwitzgebel and Cushman (2015)—nor, for that matter, the opposing result of Kühberger and Gradl (2013).

Next, we examined the relations among frame-consistent choice, strength of preference, and the four cognitive-style measures. **Table 1** shows the zero-order (Pearson) correlations. The cognitive-style measures were positively correlated with each other. Frame-consistent choice was significantly correlated with CRT in the predicted direction, but not with any other measure. However, recall that CRT differed across frame. The partial correlation between CRT and frame-consistent choice controlling for frame was not significant, *r*(187) = 0.11, *p* = 0.12.

**Table 1:** Pearson correlation matrix (Experiment 1).

Variables	1	2	3	4	5	6
1. Frame-consistent choice	1	-0.06	0.16*	-0.04	0.12	0.05
2. Preference strength		1	-0.21*	0.07	0.05	-0.03
3. CRT			1	0.27**	0.29**	0.26**
4. SNS				1	0.21**	0.39**
5. AOT					1	0.32**
6. NFC						1


*^∗^*p* < 0.05, ^∗∗^*p* < 0.01, two-tailed.*

We followed up the initial correlational analysis by running a binary-logistic regression analysis testing three models. Model 1 includes only the fixed effect (frame), Model 2 further includes the cognitive-style measures, and Model 3 further includes the control variables, sex and age. As **Table 2** shows, the only significant predictor in each of the three models was frame. This result is explained by the fact that the proportion of participants making frame-consistent choices was much larger in the positive-frame condition (0.75) than in the negative-frame condition (0.49). The one-parameter (frame) model was significant, χ^2^(1, *N* = 190) = 13.19, Nagelkerke *R*^2^ = 0.09, *p* < 0.001. Model 2 did not significantly improve fit, χ^2^(4, *N* = 190) = 7.13, Nagelkerke *R*^2^ = 0.14, *p* = 0.13. Likewise, Model 3 did not improve fit despite an effect of SNS that was almost significant, χ^2^(2, *N* = 190) = 1.38, Nagelkerke *R*^2^ = 0.15, *p* = 0.50.

**Table 2:** Binary logistic regression models predicting frame-consistent choice (Experiment 1).

					95% CI Exp (B)			
							
Model	Source	*B*	*SE*	Exp(B)	LB	UB	Wald	*p*
1	Constant	-1.16	0.48	0.31	–	–	5.90	0.015
1	Frame	1.12	0.31	3.05	1.66	5.62	12.79	0.000
2	Constant	-2.01	1.35	0.13	–	–	2.20	0.138
2	Frame	1.11	0.33	3.03	1.60	5.71	11.63	0.001
2	CRT	0.26	0.18	1.30	0.90	1.85	1.99	0.159
2	SNS	-0.31	0.19	0.73	0.50	1.06	2.68	0.102
2	AOT	0.30	0.21	1.35	0.89	2.05	1.97	0.160
2	NFC	0.09	0.19	1.09	0.75	1.59	0.21	0.644
3	Constant	-0.90	1.66	0.41	–	–	0.30	0.587
3	Frame	1.10	0.33	3.01	1.59	5.71	11.44	0.001
3	CRT	0.20	0.19	1.23	0.84	1.78	1.14	0.285
3	SNS	-0.34	0.20	0.71	0.49	1.04	3.05	0.081
3	AOT	0.31	0.22	1.36	0.89	2.08	1.98	0.159
3	NFC	0.15	0.20	1.16	0.78	1.71	0.52	0.472
3	Sex	-0.34	0.40	0.71	0.33	1.54	0.76	0.385
3	Age	-0.03	0.04	0.97	0.90	1.05	0.66	0.417


*CI, confidence interval; LB, lower bound; UB, upper bound.*

Next, we examined whether preference-weighted frame-consistent choices showed consistent results. Mean preference-weighted choice differed significantly from a test value of zero (as expected by chance) in the frame-consistent direction, *M* = 1.09, 95% CI [0.40, 1.80], one-sample *t*(189) = 3.13, *d* = 0.23, *p* = 0.002. The effect size is comparable to that found in the earlier analysis of unweighted frame-consistent choice. Moreover, this effect size based on the conservative test is, once again, substantially smaller than that obtained by the usual between-groups method. We find a mean value of 0.16 (*SD* = 4.95) in the negative-frame condition and a mean value of 2.28 (*SD* = 4.43) in the positive-frame condition, *t*(188) = 3.10, *d* = 0.45, *p* = 0.002. Finally, consistent with the earlier results of the order-effect analysis on unweighted choice, there was no significant effect of frame-consistent option order on preference-weighted choice, *t*(188) = 0.97, *d* = 0.14, *p* = 0.34.

Finally, we tested a bootstrap multiple linear regression model with frame, NFC, AOT, CRT, SNS, sex, and age as predictors of weighted frame-consistent choice. Model 1 includes only the fixed effect (frame), Model 2 further includes the cognitive-style measures, and Model 3 further includes the control variables, sex and age. The last column of **Table 3** shows that the variance inflation factors (VIFs) are all close to 1, which indicates that the interpretability of the models is not threatened by multicollinearity. **Table 3** shows that, as with the binary-choice measure, only the effect of frame was significant, Model 1 *F*(1, 188) = 12.82, adjusted *R*^2^ = 0.06, *p* < 0.001. Model 2 did not significantly improve fit, *F*_change_(4, 184) = 1.48, *p* = 0.21. Likewise, Model 3 did not improve upon the fit of Model 2, *F*_change_(2, 182) = 0.73, *p* = 0.48. The results are therefore highly consistent between analyses of binary and preference-weighted choices.

**Table 3:** Multiple linear regression models predicting preference-weighted frame-consistent choice (Experiment 1).

					95% CI			
							
Model	Source	β	*B*	*SE*	LB	UB	*p*	VIF
1	Constant	–	-2.60	1.13	-4.76	-0.24	0.026	–
1	Frame	0.25	2.44	0.67	1.07	3.73	0.001	1.00
2	Constant	–	-4.74	3.05	-11.21	1.24	0.134	–
2	Frame	0.25	2.38	0.67	0.96	3.69	0.001	1.05
2	CRT	0.08	0.36	0.37	-0.42	1.15	0.324	1.20
2	SNS	-0.12	-0.64	0.40	-1.43	0.16	0.105	1.24
2	AOT	0.11	0.68	0.49	-0.37	1.64	0.160	1.19
2	NFC	0.04	0.22	0.45	-0.63	1.17	0.628	1.28
3	Constant	–	-2.35	3.84	-10.85	4.87	0.543	–
3	Frame	0.24	2.36	0.68	0.99	3.60	0.001	1.05
3	CRT	0.05	0.24	0.38	-0.58	1.02	0.528	1.29
3	SNS	-0.13	-0.69	0.40	-1.44	0.10	0.089	1.26
3	AOT	0.11	0.64	0.50	-0.45	1.69	0.194	1.21
3	NFC	0.06	0.29	0.46	-0.63	1.26	0.549	1.38
3	Sex	-0.09	-0.95	0.82	-2.50	0.64	0.257	1.12
3	Age	-0.03	-0.03	0.10	-0.26	0.14	0.731	1.11


*All estimates except the standardized regression coefficients are based on 1,000 bias-corrected and accelerated bootstrap samples.*

## Experiment 2

The aim of Experiment 2 was to assess the reproducibility of findings from Experiment 1. In most respects, the methods of Experiment 2 were identical to Experiment 1, except that a larger sample of participants was recruited from Amazon Mechanical Turk, and we added one additional cognitive-style measure, Zenhausern’s Preference Test (ZPT, Morton, 2002). ZPT was developed in the 1970s as a measure of hemispheric dominance. The 20-item test includes 10 “right hemisphere” (ZPT-R) and 10 “left hemisphere” (ZPT-L) items, with a hemisphericity index scored as the difference of the two subscales (i.e., ZPT-R – ZPT-L). Higher ZPT index values have been interpreted as being indicative of a cognitive disposition toward the use of intuitive System 1 reasoning processes. In particular, McElroy and Seta (2003) found that participants in the highest quartile on the index showed much stronger framing effects than those in the lowest quartile, and they interpreted their findings as supporting the view that frame susceptibility is a cognitive bias owing to reliance on intuitive System 1 reasoning processes. To the best of our knowledge, however, no other study has examined whether this measure predicts frame susceptibility.

### Materials and Methods

A sample of 323 Amazon Mechanical Turk (MTurk) workers participated in Experiment 2. The sample was limited to participants who were 18 years of age or older, residing in Canada or the United States, and who completed greater than or equal to 1,000 Human Intelligence Tasks or “HITs” with an approval rate greater than or equal to 95%. Participants were compensated $1.50. Mean age for the sample was 30.4 years (*SD* = 6.7), and 39.2% were female. Seven participants were removed from the analysis because of either (a) unreasonable time for completion (over 24 h, or under 4 min) or (b) reported English proficiency was below 50%. Data from the remaining 316 participants were analyzed.

Except for the change of sample and inclusion of ZPT in the battery of cognitive-style measures, the methods were identical to Experiment 1. Characteristics of the cognitive-style measures were as follows: ZPT-L: *M* = 7.36, *SD* = 1.03, Cronbach’s alpha = 0.70; ZPT-R: *M* = 5.86, *SD* = 1.46, Cronbach’s alpha = 0.82; CRT: *M* = 2.06, *SD* = 1.16, Cronbach’s alpha = 0.80; SNS: *M* = 4.66, *SD* = 0.83, Cronbach’s alpha = 0.83; AOT: *M* = 5.37, *SD* = 0.93, Cronbach’s alpha = 0.80; NFC: *M* = 5.98, *SD* = 1.62, Cronbach’s alpha = 0.95. The ZPT hemisphericity index was computed by subtracting ZPT-L from ZPT-R. All scale reliabilities were good (i.e., >0.70), and invariably greater than in Experiment 1, perhaps reflecting the change of sample from undergraduates to MTurk workers. Finally, none of the measures differed significantly across frame (*p*s > 0.11).

### Results and Discussion

Out of 316 participants, 184 (58.2%) made frame-consistent choices. As in Experiment 1, this proportion significantly exceeds chance: the binomial probability of finding 184 or more frame-consistent choices is 0.002. As noted earlier, the expected value based on chance selection is 0, and the observed mean value is 0.16 (*SD* = 0.99). This corresponds to a small effect size, *d* = 0.17, which is very close in magnitude to that found in Experiment 1. By comparison, using the traditional between-groups analysis, we find a mean value of -0.03 (*SD* = 1.00) in the negative-frame condition and a mean value of 0.30 (*SD* = 0.96) in the positive-frame condition, *t*(314) = 3.00, *d* = 0.34, *p* = 0.003. Therefore, by applying the stricter, theory-constrained, criterion, the effect size was once again substantially reduced (namely, halved).

As in Experiment 1, no order effect was found: the proportion of frame-consistent choices when the frame-consistent option was presented first (0.65) was not significantly greater than the proportion of such choices when the frame-consistent option was presented second (0.55), *p* = 0.31 by Fisher’s two-sided exact test, Goodman and Kruskal τ = 0.004.

As **Table 4** shows, and replicating the findings of Experiment 1, frame-consistent choice was not significantly correlated with strength of preference or any of the cognitive-style measures. We followed up the zero-order correlational analysis by running a binary logistic regression analysis. As in Experiment 1, we tested three models: Model 1 included frame only, Model 2 additionally included CRT, SNS, AOT, NFC, and ZPT, and Model 3 additionally included sex and age. As **Table 5** shows, the only significant predictor of frame-consistent choice was frame. As in Experiment 1, the proportion of participants making frame-consistent choices was larger in the positive-frame condition (0.65) than in the negative-frame condition (0.52). The one-parameter (frame) model was significant, χ^2^(1, *N* = 316) = 5.85, Nagelkerke *R*^2^ = 0.03, *p* = 0.02. Model 2 did not significantly improve fit, χ^2^(5, *N* = 316) = 8.89, Nagelkerke *R*^2^ = 0.06, *p* = 0.11. Likewise, Model 3 did not improve fit over Model 2 in spite of effects of ZPT and age that were almost significant, χ^2^(2, *N* = 316) = 3.16, Nagelkerke *R*^2^ = 0.07, *p* = 0.21.

**Table 4:** Pearson correlation matrix (Experiment 2).

Variables	1	2	3	4	5	6	7
1. Frame-consistent choice	1	0.04	-0.02	-0.04	0.08	0.10	0.09
2. Preference strength		1	-0.11	-0.01	-0.05	-0.01	0.02
3. CRT			1	0.27**	0.32**	0.19**	-0.22**
4. SNS				1	0.29**	0.32**	-0.10
5. AOT					1	0.34**	-0.20**
6. NFC						1	0.11
7. ZPT							1


*^∗^*p* < 0.05, ^∗∗^*p* < 0.01, two-tailed.*

**Table 5:** Binary logistic regression models predicting frame-consistent choice (Experiment 2).

					95% CI Exp (B)			
							
Model	Source	*B*	*SE*	Exp (B)	LB	UB	Wald	*p*
1	Constant	-0.49	0.36	0.61	–	–	1.88	0.170
1	Frame	0.56	0.23	1.74	1.11	2.74	5.79	0.016
2	Constant	-0.96	0.87	0.38	–	–	1.20	0.273
2	Frame	0.54	0.24	1.71	1.08	2.71	5.19	0.023
2	CRT	-0.02	0.11	0.98	0.79	1.22	0.02	0.876
2	SNS	-0.21	0.15	0.81	0.61	1.08	2.01	0.156
2	AOT	0.19	0.14	1.21	0.92	1.61	1.80	0.180
2	NFC	0.11	0.08	1.12	0.96	1.31	1.92	0.166
2	ZPT	0.13	0.08	1.14	0.97	1.35	2.58	0.108
3	Constant	-0.52	1.07	0.59	–	–	0.24	0.625
3	Frame	0.56	0.24	1.74	1.10	2.78	5.50	0.019
3	CRT	0.00	0.11	1.00	0.80	1.24	0.00	0.998
3	SNS	-0.20	0.15	0.82	0.62	1.10	1.73	0.188
3	AOT	0.23	0.15	1.26	0.94	1.67	2.43	0.119
3	NFC	0.12	0.08	1.12	0.96	1.32	2.02	0.155
3	ZPT	0.15	0.09	1.16	0.98	1.37	2.98	0.084
3	Sex	0.14	0.25	1.15	0.71	1.87	0.33	0.564
3	Age	-0.03	0.02	0.97	0.94	1.01	2.91	0.088


*CI, confidence interval; LB, lower bound; UB, upper bound.*

Next, we examined whether preference-weighted choices showed consistent results. Mean preference-weighted choice differed significantly from a test value of zero in the frame-consistent direction, *M* = 0.90, 95% CI [0.32, 1.42], one-sample *t*(315) = 3.05, *d* = 0.17, *p* = 0.002. The effect size for the weighted measure was identical to what we reported earlier for the unweighted measure. Moreover, this effect size based on the conservative test is, once again, substantially smaller than that obtained by the between-groups method. We find a mean value of 0.09 (*SD* = 5.09) in the negative-frame condition and a mean value of 1.89 (*SD* = 5.07) in the positive-frame condition, *t*(314) = 3.13, *d* = 0.35, *p* = 0.002. Finally, showing strong consistency with the results of Experiment 1, there was no significant effect of frame-consistent option order on preference-weighted choice, *t*(314) = 1.05, *d* = 0.12, *p* = 0.30.

Finally, we tested a bootstrap multiple linear regression model with frame, CRT, SNS, AOT, NFC, ZPT, sex, and age as predictors of weighted frame-consistent choice. The model structure for this analysis was identical to that tested in Experiment 1. As the VIF values shown in the last column of **Table 6** indicate, the interpretability of the models is not threatened by multicollinearity. As **Table 6** shows, Model 1, which includes only frame as a predictor, was significant, *F*(1, 314) = 11.77, adjusted *R*^2^ = 0.03, *p* = 0.001. Frame was once again significant in Model 2, and ZPT approached significance, as did the Model 2 improvement of fit, *F*_change_(5, 309) = 2.04, adjusted *R*^2^ = 0.05, *p* = 0.073. Model 3 did not improve upon the fit of Model 2, *F*_change_(2, 307) = 1.55, *p* = 0.22. As in Experiment 1, then, the results were highly consistent between analyses of participants’ binary and preference-weighted choices.

**Table 6:** Multiple linear regression models predicting preference-weighted frame-consistent choice (Experiment 2).

					95% CI			
							
Model	Source	β	*B*	*SE*	LB	UB	*p*	VIF
1	Constant	–	-2.07	0.88	-3.81	-0.30	0.021	–
1	Frame	0.19	1.98	0.56	0.86	3.00	0.001	1.00
2	Constant	–	-3.62	2.18	-7.99	1.15	0.103	–
2	Frame	0.18	1.90	0.56	0.72	2.98	0.001	1.02
2	CRT	-0.02	-0.08	0.27	-0.63	0.44	0.725	1.21
2	SNS	-0.07	-0.38	0.38	-1.04	0.29	0.309	1.21
2	AOT	0.07	0.43	0.37	-0.31	1.12	0.246	1.31
2	NFC	0.10	0.31	0.22	-0.09	0.76	0.153	1.27
2	ZPT	0.11	0.39	0.22	-0.04	0.79	0.075	1.13
3	Constant	–	-2.81	2.65	-8.01	2.49	0.298	–
3	Frame	0.18	1.92	0.56	-0.77	2.99	0.001	1.02
3	CRT	-0.01	-0.04	0.27	-0.58	0.47	0.896	1.21
3	SNS	-0.06	-0.34	0.38	-1.01	0.39	0.367	1.22
3	AOT	0.09	0.51	0.37	-0.22	1.19	0.168	1.33
3	NFC	0.10	0.32	0.22	-0.11	0.78	0.150	1.27
3	ZPT	0.12	0.41	0.22	-0.00	0.82	0.062	1.14
3	Sex	0.04	0.46	0.61	-0.77	1.63	0.455	1.05
3	Age	-0.09	-0.07	0.04	-0.15	0.02	0.073	1.04


*All estimates except the standardized regression coefficients are based on 1,000 bias-corrected and accelerated bootstrap samples.*

## General Discussion

The two online experiments that we conducted yielded highly consistent results despite the fact that one experiment relied on a university undergraduate sample where participants received course credit and the other experiment relied on a MTurk worker sample whose members were paid a nominal rate for their participation. Although it would be advantageous to attempt to replicate the findings in experiments in which participants were not completing the experiment remotely online and in which other variants of the ADP-type task were used, we believe several of the present findings are nevertheless noteworthy.

First, in both experiments we observed levels of frame-consistent decision-making that are unlikely to be due to chance. We find evidence of frame susceptibility even when steps are taken to rule out linguistic interpretations of the options, such as lower bounding of numerical quantifiers, which would invalidate the assumption of extensional equivalence of alternatively framed options. These task design features, which promote clearer interpretability of data from ADP-type tasks (Mandel, 2014), might account for the somewhat lower effect size observed using the conventional between-groups measure. The meta-analytic (framing) effect size for the two experiments reported here is *d* = 0.42, 95% CI [0.22, 0.62]. The meta-analytic effect size from 80 ADP-type studies was 0.57 (Kühberger, 1998). Thus, while lower, the meta-analytic effect size in the present studies is only marginally so.

At first blush, the present findings may appear to contradict those reported by Mandel (2014, Experiment 2). In that experiment, when the term *exactly* was used to prompt a bilateral interpretation of the numerical quantifiers, no significant framing effect was observed. To compare that effect in terms of statistical significance, however, would be misleading because the sample size for that experimental condition was 76, whereas the present experiments matching that condition collectively sampled over 500 participants. Therefore, the present research had much greater statistical power to detect small effects. Drawing on the raw data from the earlier experiment, 44 of the 76 participants (i.e., 57.9%) made frame-consistent choices. The binomial probability of obtaining that number or greater is 0.103. However, the proportion obtained in Mandel (2014, Experiment 2) does not significantly differ from the proportion obtained in Experiments 1 and 2 of the present research. The difference in proportions in the former case (Experiment 1) is 0.042, 95% CI [-0.084, 0.017], and in the latter case (Experiment 2) it is 0.003, 95% CI [-0.115, 0.127]. In other words, quite to the contrary, there is strong consistency in the results, which indicate the existence of a framing effect of small magnitude that may or may not be statistically significant depending on sample size in ADP-type decision-making tasks.

A second noteworthy finding was that no significant effect of option order on choice was found in either experiment. Nevertheless, in each experiment, the proportion of choices that were frame-consistent was greater when the frame-consistent choice was presented first. These non-significant differences are in the same direction as that reported by Schwitzgebel and Cushman (2015), and also in line with the reachability bias (Bar-Hillel et al., 2014). Moreover, if we combine our samples, the effect approaches significance using a one-tailed test: 63.1% choose the frame-consistent choice when that option was presented initially versus 56.1% when that option was presented last, *p* = 0.057 by Fisher’s exact one-sided test. Thus, our findings provide faint evidence in support of reachability bias in the context of framing tasks. Although the effect of option order on choice in ADP-type tasks appears to be very weak, it nevertheless should be experimentally controlled (at minimum, through counterbalancing) in future research.

A key finding of this research was that cognitive-style measures had very small predictive effects on frame susceptibility. All were non-significant in each experiment, although there was a small zero-order correlation between CRT and frame susceptibility in Experiment 1 that explained approximately 2.5% of the variance. Taken together, these findings do not support the hypothesis that individual differences in frame susceptibility in decision-making are substantially due to differences in cognitive style—or more specifically, in the degree to which people choose intuitively or deliberately. Moreover, if the true relation between cognitive-style measures and frame susceptibility is weak in the general population, we would expect to see a pattern of results much like we observe in the literature; namely, one in which there appears to be “mixed evidence” in which some studies find significant (but weak) relations and other studies find non-significant relations (that are weak but usually in the expected direction).

Such evidence is “mixed” only in a trivial sense—namely, when researchers pay undue attention to statistical significance across studies that vary in statistical power. The significant effects of cognitive style on frame susceptibility in ADP-type tasks that have been reported in the literature are in most cases small, even when large samples have been used to boost the likelihood of detecting a significant effect (e.g., West et al., 2008). Those results, moreover, are in line with other findings showing that the effect of cognitive ability on judgment and decision-making tasks used to demonstrate cognitive biases and use of heuristic processes is small (e.g., Stanovich and West, 2008; West et al., 2012). The true magnitude of the effect of cognitive style (gauging the System 1/System 2 distinction) on frame susceptibility is therefore likely to lie somewhere between very small and small, using Cohen’s (1992) criteria. The precise value is theoretically unimportant because the range is sufficient to indicate that any theory positing that framing effects are largely due to reliance on heuristic “System 1” reasoning processes is wrong. Of course, we do not carelessly generalize this claim to other judgment and decision-making tasks. We acknowledge that there is good evidence that measures of thinking style predict performance on some judgment and decision-making tasks that have been used to demonstrate cognitive biases (e.g., Stanovich and West, 2000). However, this proviso cuts both ways, and we believe researchers should be circumspect in including ADP-type tasks as items in aggregated measures of cognitive bias (e.g., Bruine de Bruin et al., 2007; Toplak et al., 2011).

Critics might counter that we have not made the most of our data by examining the relations between frame-consistent choice and the cognitive-style measures in the combined sample. If we found small but significant effects that would of course reinforce rather than challenge our conclusion. In fact, even with the combined sample of 506 participants, the zero-order correlations between frame-consistent choice and the cognitive-style measures were invariably not statistically significant and the correlations were all close to nil (*r*s = 0.02, -0.05, 0.08, and 0.08 for CRT, SNS, AOT, and NFC, respectively). Clearly, the results are not due to a lack of statistical power.

Critics might also charge that we have not gone far enough in exploring the possible predictive utility of the measures we investigated. For instance, it is conceivable that CRT would show a stronger relation to frame susceptibility if it were scored in terms of whether the typical intuitive response was selected rather than whether the correct response was selected (Pennycook and Ross, 2016; Stupple et al., 2017). However, this was not the case. If we sum the number of intuitive responses, the correlation remains small in Experiment 1 (*r* = -0.14, *p* = 0.056) and it is virtually nil in Experiment 2 (*r* = 0.01, *p* = 0.80). Nor does an item-response analysis of CRT alter our conclusions. The largest correlation obtained between frame susceptibility and whether or not responses to an item were intuitive was -0.14 (for the lily-pad problem in Experiment 1).

Another possible line of investigation would be to treat the scales as items and to extract factor scores that might prove to be more highly correlated with frame susceptibility. To explore this, we factor analyzed the four measures common to both experiments (CRT, SNS, AOT, and NFC) separately within each experiment. In both cases, using principal components analysis with varimax rotation, a single factor had initial Eigenvalues greater than 1. The factor scores were not significantly correlated with frame susceptibility in either experiment: in Experiment 1, *r* = 0.10 (*p* = 0.18), and in Experiment 2, *r* = 0.05 (*p* = 0.41). Therefore, we find very weak evidence—even using a variety of analytic and data-pooling techniques—to support the hypothesis that individual differences in frame susceptibility are well accounted for by individual differences in thinking style or disposition. To the contrary, the multi-measure, multi-method approach used in this research strongly supports the alternative hypothesis that frame susceptibility in decision-making is not substantially explained by the facets of cognitive style that we examined.

Only one factor explained variation in frame susceptibility in the two experiments and that was the framing manipulation itself. Participants were more likely to make frame-consistent choices in the positive-frame condition than in the negative-frame condition. We strongly suspect that this result is due to a tendency toward risk aversion in the present experiments. This finding is consistent with literature showing that decision-making tasks involving representations of human life (like the ADP) tend to elicit risk-seeking choices, whereas problems with comparable deep structure that instead involve financial outcomes (such as the ADP variant used in the present research) tend to elicit risk-averse choices (e.g., see Jou et al., 1996; Wang, 1996; Fagley and Miller, 1997), perhaps due to the higher aspiration levels set in the morally charged life domain (Schneider, 1992; Rettinger and Hastie, 2003). Hence, the effect of frame on frame susceptibility is likely to be predictable on the basis of content effects on decision-making (Wagenaar et al., 1988; Mandel and Vartanian, 2011). Such content effects, in turn, are likely to be moderated by other decision-task characteristics, such as the payoff structure of choices. In ADP-like problems, a failure to choose would result in maximum sure loss. Clearly (and fortunately), not all decisions are like this. In tasks in which participants must choose between certain and uncertain options but in which inaction implies the status quo, there tends to be greater risk aversion for human-life problems than for monetary problems (Vartanian et al., 2011).

Current theories of framing are not well adapted to explaining such content effects. As noted earlier, most theories of framing make comparable predictions in the ADP—namely, choice of the certain option under positive framing and choice of the uncertain option under negative framing based on inflexible psychophysical assumptions as captured in the stylized value function of prospect theory (Kahneman and Tversky, 1979) or equally inflexible linguistic assumptions as captured in the transformation rules of fuzzy trace theory (Reyna and Brainerd, 1991; Chick et al., 2016). The explicated valence account—or EVA (Tombu and Mandel, 2015), which elucidates how frames (through their explication of outcome valence) affect representations of risk, is more conducive to accommodating content and task effects because the latter, too, appear to influence decision-making through altering risk perceptions. However, EVA currently does not explicitly integrate such factors and would thus require further development.

Much the same could also be said of the editing phase in prospect theory, which is essentially a representational pre-processing stage of decision-making. It is noteworthy that early theoretical attention to framing effects focused on the value function in prospect theory, which predicts risk aversion in the domain of gain and loss aversion in the domain of loss (where the domains are separated by a neutral reference point). Yet, several decades on, it now appears that frames affect the manner in which aspects of problems are mentally represented (Mandel, 2008). The representational effects not only include reference-point selection, as Tversky and Kahneman (1981) had surmised, but also representation of intended communication (e.g., Sher and McKenzie, 2008; van Buiten and Keren, 2009; Teigen, 2011), quantity and probability (Mandel, 2014), and option risk (Tombu and Mandel, 2015). The effects of alternative frames on such representations are probabilistic and naturally give rise to individual differences in representation. For instance, whereas a majority of participants adopted a lower-bound (“at least”) interpretation of the certain options in the standard ADP, nearly one-third adopted a bilateral (“exactly”) interpretation of the same options (Mandel, 2014, Experiment 3). Surprisingly little research attention has been given to exploring these representational effects. Given how weakly cognitive-style measures predict individual differences in frame susceptibility, research attention to the representational consequences of framing could shed important light on the bases for such individual differences.

## Author Contributions

DM and IK developed the experiments and analyzed the data. IK executed the experiments. IK contributed to the writing of the manuscript, which was primarily written by DM.

## Conflict of Interest Statement

The authors declare that the research was conducted in the absence of any commercial or financial relationships that could be construed as a potential conflict of interest.

## Footnotes

1 Although, we are aware of Knight’s (1964) distinction between risk and uncertainty, we prefer to use the term *uncertain* rather than *risky* for options B and D in the ADP because (a) Mandel (2014) has shown that participants often have imprecise interpretations of ostensibly precise probabilities such as “1/3 chance.” Moreover, Tombu and Mandel (2015) have shown that a non-trivial proportion of participants perceive option C as riskier than option D.

2 West et al. (2012) examined the relations between NFC, AOT, and CRT and a composite measure of responses from judgment and decision-making tasks associated with cognitive biases. These tasks included the ADP, but unfortunately, their analyses do not extend to the specific relations between the cognitive-style measures and the ADP. Their findings, however, indicate that all three measures showed small correlations (˜0.1) with the composite measure of cognitive biases.

## Supplementary Material

DATA SHEET S1 Data for Experiments 1 and 2.
